# A Review and Tutorial of Machine Learning Methods for Microbiome Host Trait Prediction

**DOI:** 10.3389/fgene.2019.00579

**Published:** 2019-06-25

**Authors:** Yi-Hui Zhou, Paul Gallins

**Affiliations:** ^1^Department of Biological Sciences, North Carolina State University, Raleigh, NC, United States; ^2^Bioinformatics Research Center, North Carolina State University, Raleigh, NC, United States

**Keywords:** disease, phenotype, modeling, machine learning, prediction

## Abstract

With the growing importance of microbiome research, there is increasing evidence that host variation in microbial communities is associated with overall host health. Advancement in genetic sequencing methods for microbiomes has coincided with improvements in machine learning, with important implications for disease risk prediction in humans. One aspect specific to microbiome prediction is the use of taxonomy-informed feature selection. In this review for non-experts, we explore the most commonly used machine learning methods, and evaluate their prediction accuracy as applied to microbiome host trait prediction. Methods are described at an introductory level, and R/Python code for the analyses is provided.

## 1. Introduction

The microbiome is the collection of all microbes living in or on a host, including bacteria, viruses, and fungi (Robinson and Pfeiffer, 2014). The risk or severity of numerous diseases and disorders in a host are associated with the microbiome (Kinross et al., 2011), and accurate trait prediction based on microbiome characteristics is an important problem (Rothschild et al., 2018). The application of modern machine learning algorithms is proving to be valuable in this effort (Gilbert et al., 2018). This review/tutorial focuses on the bacterial component of the microbiome, although in principle many of the elements apply more generally.

With modern high-throughput sequencing, entire microbial communities can be profiled, revealing an extensive diversity of genes and organisms (Turnbaugh et al., 2007). A common strategy is to sequence only a highly specific region, such as 16S ribosomal RNA (rRNA), although the methods described below can also be applied to metagenomic shotgun methods (Mande et al., 2012). Due to the graded nature of sequence similarity, the data are often organized into operational taxonomic units (OTUs) (Schmitt et al., 2012), i.e., clusters of similar sequences, intended to represent the abundance of a particular bacterial taxon while avoiding excessive sparsity that would result if only identical sequences were grouped. Typical choices of similarity limits (e.g., grouping sequences with no more than 3% dissimilarity) produce taxa that are specific to bacterial species, or represent a further subdivision within species. Informatic methods for taxonomic classification use databases (McDonald et al., 2012), such as SILVA (Quast et al., 2012), and are beyond our scope, but we assume that such classification is available. The result after OTU grouping is a matrix (OTU table) of OTU features by the number of samples, where the number of features can vary dramatically across datasets due to stringency of grouping. Although methods that avoid OTU grouping have been described (Callahan et al., 2016), OTU tables remain common and are a practical starting point for most machine learning prediction methods. For additional discussion of levels of taxonomy, with intriguing thoughts about the interplay and use of molecular function descriptors vs. taxonomic descriptors, the reader is referred to Knights et al. (2011b) and Xu et al. (2014). However, many of the principles discussed here apply regardless of the feature type.

Several features of OTU tables present challenges. First, OTU tables are sparse, with a large proportion of zero counts (Hu et al., 2018). Investigators have often removed OTUs that were present in too few samples to be useful, or collapsed OTUs into the genus level, which is a simple form of “feature engineering” that we will explore further below. Second, the role of taxonomy in prediction is often unclear – similar sequences are often correlated across samples, which is a property that can be readily assessed directly without taxonomic knowledge. Third, as with many omics technologies, library sizes (essentially column sums of the OTU table) vary considerably, and normalization methods must be used to account for this variation (Weiss et al., 2017).

A number of excellent reviews have been published, covering experimental design and targeted amplicon vs. metagenomics profiling (Mallick et al., 2017), and a comprehensive overview of different experimental and interrogation methods and analyses (Knight et al., 2018). Other reviews have covered the remarkable advances in understanding that have resulted recently in understanding connections of, e.g., human gut microbiome populations to human health (Cani, 2018).

Recently, studies have begun to explore the power of machine learning to use microbiome patterns to predict host characteristics (Knights et al., 2011a; Moitinho-Silva et al., 2017). Existing studies often report disease-associated dysbiosis, a microbial imbalance inside the host, but such associations can have a wide range of interpretations. Individual studies have also suffered from small sample sizes, inconsistent findings, and a lack of standard processing and analysis methods (Duvallet et al., 2017). Prediction models have sometimes been difficult to generalize across studies (Pasolli et al., 2016). One approach to resolve these issues is by performing a meta-analysis, combining microbiome studies across common traits. Duvallet et al. (2017) have performed a cross-disease meta-analysis of published case-control gut microbiome studies spanning 10 diseases. They found consistent patterns characterizing disease-associated microbiome changes and concluded that many associations found in case-control studies are likely not disease-specific but rather part of a non-specific, shared response to health and disease. Pasolli et al. (2016) also performed a meta-analysis in a collection of 2,424 publicly available samples from eight large-scale studies. The authors remarked that addition of healthy (control) samples from other studies to training sets improved disease prediction capabilities. Nonetheless, any meta- or pooled analysis should rely on a solid foundation of effective per-study prediction. The use of multiple studies enabled Pasolli et al. (2016) to explore the use of external validation of models across truly separate datasets. Such external validation can in principle result in more robust and generalizable models for prediction than models that are validated internally only.

Sophisticated machine learning methods in microbiome analysis have been proposed considerably in recent years, including using deep neural networks (Ananthakrishnan et al., 2017), and leveraging methods for genomes and metagenomes (Rahman et al., 2018). However, the content-knowledge required to implement these methods is high, presenting a barrier to data scientists looking to get started in microbiome analysis and prediction. Moreover, there are few resources for biologists with intermediate statistical and computing background to “jump in” to analysis of the important trait prediction problem. The target audience of this paper is those seeking a brief review and tutorial for trait prediction, and who will benefit from accessible code. After digesting these basic building blocks of analysis, the reader may move to more advanced, such as dynamic systems modeling (Brooks et al., 2017).

The remainder of this paper is written in several sections. Section 2 reviews the steps of data preparation before machine learning implementation. Section 3 provides a quick overview of the most commonly-used machine learning (ML) methods, as well as the most commonly used performance criteria. Experienced modelers can skip this section. Section 4 summarizes the scope of the relevant literature and describes several real datasets and the trait of interest. Section 5 provides results, and the underlying code forms a tutorial of machine learning methods applied in this context.

## 2. Data Preparation

Many machine learning methods have difficulty with missing features, and so we assume the OTU table is complete. A minor fraction of missing data can often be effectively handled using simple imputation procedures, such as kNN-impute (Crookston and Finley, 2008), or even simpler methods, such as feature-median imputation. The methods described in this section, including imputation and normalization, must be performed without using the host trait information, because otherwise they might be biased by this information. Feature selection methods that use host trait information belong in the next section, as they must be included inside a cross-validation procedure.

### 2.1. Notation and Sampling Considerations

Let *X* be an *m* × *n* matrix of microbiome count data, where *m* is the number of OTU features and *n* is the number of samples. Let *y* be a vector of length *n* with the microbiome host trait. Commonly a trait will be a binary outcome (e.g., case/control status, coded 1/0), or a continuous trait, such as body mass index (BMI). Here our use of microbiome features as predictive of a trait does not imply or assume causality. We note that case/control study designs often involve oversampling of one type (often cases) relative to the general population. A prediction rule might explicitly use this information, for example by a simple application of Bayes' rule (Tibshirani et al., 2003), with prior probabilities reflecting those in the general population. Such sampling considerations are beyond our scope, and we refer the reader to Chawla (2009). Here we consider our sample dataset to be representative of the population of its intended downstream use.

### 2.2. Transformation and Normalization

Normalization is an essential process to ensure comparability of data across samples (Weiss et al., 2017), largely to account for the large variability in library sizes (total number of sequencing reads across different samples). The basic issues are similar to those encountered in expression sequence normalization (de Kok et al., 2005), but less is currently known about sources of potential bias to inform microbiome normalization. Normalization methods assessed by Weiss et al. (2017) included cumulative sum scaling, variance stabilization, and trimmed-mean by *M*-values. Randolph et al. (2018) utilized the centered log-ratio (CLR) transform of the relative abundance vectors, based on a method developed by Aitchison (1982), replacing zeros with a small positive value. As part of their motivation, Randolph et al. (2018) pointed out that standard cumulative sum scaling places the normalized data vectors in a simplex, with potential consequences for kernel-based discovery methods (Randolph et al., 2018).

### 2.3. Taxonomy as Annotation

Taxonomy is the science of defining and naming groups of biological organisms on the basis of shared characteristics. In our context, taxonomy refers to the evolutionary relationship among the microbes represented by each OTU, from general to specific: kingdom, phylum, class, order, family, genus and species, and OTU (Oudah and Henschel, 2018). For example, Kostic et al. (2012) summarized their findings in the study of microbiota in colorectal cancer using genera and phyla-level summaries, illustrating the importance of taxonomy in interpretation. Here we are highlighting the use of taxonomy in *post-hoc* interpretation of findings, providing important biological context. However, if the taxonomy is used in a supervised manner to improve prediction, it then becomes part of the formal machine learning procedure, as described in the next section.

## 3. Review of Machine Learning Methods for Prediction

Machine learning deals with the creation and evaluation of algorithms to recognize, classify, and predict patterns from data (Tarca et al., 2007). Unsupervised methods identify patterns apparent in the data, but without the use of pre-defined labels (traits, in our context). These methods include (i) hierarchical clustering, which builds a hierarchy of clusters using a dendrogram, combining or splitting clusters based on a measure of dissimilarity between vectors of *X*; and (ii) *k*-means clustering, which involves partitioning the *n* vectors of *X* into *k* clusters in which each observation is classified to a cluster mean according to a distance metric. Unsupervised methods are important exploratory tools to examine the data and to determine important data structures and correlation patterns.

For the host trait prediction problem, we focus on supervised methods, in which labels (traits) of a dataset are known, and we wish to train a model to recognize feature characteristics associated with the trait. A primary difficulty in the problem is that the number of features (*m* rows) in the OTU table may greatly exceed the sample size *n*, so that over-fitting of complex models to the data is a concern.

### 3.1. Training and Cross-Validation

Training a model in supervised learning amounts to finding a parameter vector β that represents a rule for predicting a trait *y* from an *m*-vector *x*. This rule may take the form of a regression equation or other prediction rule. Prediction rules that use only a few features (*n* or fewer) are referred to as “sparse.” A good prediction rule has high accuracy, as measured by quantities, such as the area under the receiver-operator characteristic curve, or the prediction correlation *R*, both described below. Many prediction methods proceed by minimizing an objective function *obj*(β) = *L*(β) + Ω(β), which contains two parts: the raw training loss *L* and a regularization term Ω. The training loss measures how predictive the model is with respect to the data used to train the model, and the regularization term penalizes for the complexity of the model, which helps to avoid overfitting.

An essential component of machine learning is the use of cross-validation to evaluate prediction performance, and often to select tuning parameters that govern the complexity of the model. One round of *k*-fold cross-validation involves partitioning the *n* samples into *k* subsets of roughly equal size, using each subset in turn as as the validation data for testing the algorithm, with the remaining samples as the training set. After a single round of cross-validation, each sample *i* has an associated predicted trait value ŷ_*i*_, where the prediction rule was developed without any knowledge of the data from sample *i* (or at least without knowledge of *y*_*i*_). The performance measure is computed by comparing the length-*n* ŷ vector to the true *y*. To reduce variability, multiple rounds of cross-validation are performed using different partitions, and the validation results are averaged over the rounds to give an estimate of the predictive performance. Although the term “cross-validation” formally refers to the use of each sample *i* as both part of the training set and as testing set (i.e., crossing) during a single round, the term is often used more generically. For example, researchers sometimes use a simple holdout method in which a fraction 1/*k* of the data are randomly selected as a test set, the remainder as training, and repeat the process randomly with enough rounds to provide a stable estimate of accuracy.

### 3.2. Taxonomy and Structural Feature Extraction

Our Results section shows the results of prediction methods using all OTUs, as well as reduced-OTU selected or aggregated features. Several methods have been proposed to reduce the number of OTU features using correlation and taxonomy information, including Fizzy (Ditzler et al., 2015a), MetAML (Pasolli et al., 2016), and HFE (Oudah and Henschel, 2018). Aspects of the approaches are supervised and thus must be handled inside a cross-validation procedure.

For simplicity, here we focus on the hierarchical feature engineering (HFE) algorithm created by Oudah and Henschel (2018), which uses correlation and taxonomy information in *X* to exploit the underlying hierarchical structure of the feature space. The HFE algorithm consists of four steps: (1) feature engineering: consider the relative abundances of higher taxonomic units as potential features by summing up the relative abundances of their respective children in a bottom-up tree traversal; (2) correlation-based filtering: calculate the correlation of values for each parent-child pair in the taxonomy hierarchy, and if the result is greater than a predefined threshold, then the child node is discarded; (3) information gain (IG) based filtering, reflecting association of features to the trait: construct all paths from the leaves (OTUs) to the root and for each path, calculate the IG of each node with respect to the trait values, and then calculate and use the average IG as a threshold to discard any node with a lower IG score; (4) IG-based leaf filtering: for OTUs with incomplete taxonomic information, discard any leaf with an IG score less than the global average IG score of the remaining nodes from the third phase. Steps (3) and (4) must be cross-validated, as they use the trait values. The python code for implementation is on our site (https://sites.google.com/ncsu.edu/zhouslab/home/software?).

The result is a set of informative features, perhaps including original OTUs along with higher-level aggregations of taxonomic features, that can be utilized for downstream machine learning (Oudah and Henschel, 2018). Standard feature selection algorithms, Fizzy and MetAML, which do not capitalize on the hierarchical structure of features, were also tested by Oudah and Henschel (2018) using several machine learning methods on real datasets. Since HFE was reported to outperform other methods (Oudah and Henschel, 2018) and resulted in higher prediction performance overall, we apply it in the real data analysis section to extract OTU features before applying machine learning methods of trait prediction. Note that feature selection can in principle be performed inside a grand cross-validation and prediction loop, or performed prior to prediction, as we have done for convenience here.

### 3.3. Supervised Learning Methods Commonly Used in Trait Prediction

Here we list the learning methods most commonly used in microbiome host trait prediction. The list is not exhaustive, but reflects our review of the methods in common use. In particular, neural networks have received considerable recent attention, but it is difficult to find quantitative evidence for the additional predictive ability in comparison to other methods. For several of the methods, it is common to center and row-scale *X* prior to application of the method, so each feature is given similar “weight” in the analysis.

#### 3.3.1. Regression

The use of linear models enables simple fitting of continuous traits *y* as a function of feature vectors. However, if *m* ≥ *n* then structural overfitting occurs, and even if *m* < *n* accuracy is often improved by using penalized (regularized) models. For the model *y* = *Xβ* + ϵ, the training loss is $\underset{i}{\sum }{\left(y_{i}-{ŷ}_{i}\right)}^{2}$ the most commonly-used regularization methods are ridge regression (Hoerl and Kennard, 1970) and Lasso (Tibshirani, 1996) regression, which respectively use penalties $\lambda \underset{i}{\sum }\beta_{i}^{2}$ and $\lambda \underset{i}{\sum }|\beta_{i}|$ (not including the intercept) to the training loss. For binary class prediction, the approach is essentially the same, applying a generalized linear (logit) model, with the negative log-likelihood as the training loss. Here λ is a tuning parameter that can be optimized as part of cross-validation. Both methods provide “shrunken” coefficients, i.e., closer to zero than an ordinary least-squares approach. The results for Lasso are also sparse, with no more than *n* non-zero coefficients after optimization, and thus Lasso is also a feature-selection method. Another variant is the elastic net (Zou and Hastie, 2005), an intermediate version that linearly combines both penalties.

#### 3.3.2. Linear Discriminant Analysis (LDA)

**Figure 1:**
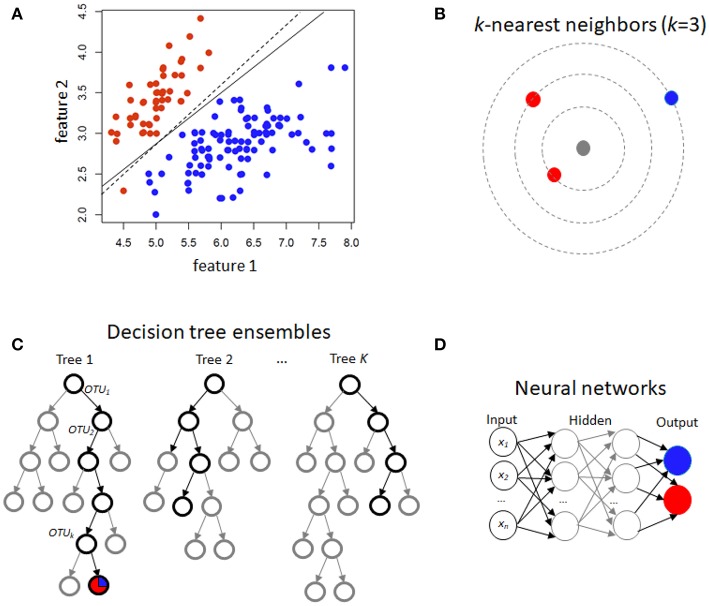
Schematic illustration of several machine learning prediction methods using case/control (red/blue) status. For two features, **(A)** illustrates linear discrimination methods. The solid line shows the linear discriminant line corresponding to equally probable outcomes, while the dashed line shows the midpoint of the maximum-margin support vector machine. **(B)** For *k*-nearest neighbors, the gray point is predicted using an average of the neighbors (red, in this instance). **(C)** Decision tree ensembles include random forests, which average over bootstrapped trees, and boosted trees, where successive residuals are used for fitting. Trees may not extend to the level of individual observations, and modal or mean values in the terminal nodes are used for prediction. **(D)** A neural network with few hidden layers.

For binary traits, this approach finds a linear combination of OTUs in the training data that models the multivariate mean differences between classes (Lachenbruch and Goldstein, 1979). Classical LDA assumes that feature data arise from two different multivariate normal densities according to *y* = 0 and *y* = 1, i.e., *MVN*(μ_0_, Σ) and *MVN*(μ_1_, Σ) (Figure 1A). The prediction value is the estimate of the posterior mean *E*(*Y*|*x*) = *Pr*(*Y* = 1|*X*), used because it minimizes mean-squared error.

#### 3.3.3. Support Vector Machines (SVM)

This is another approach in the linear classifier category (Figure 1A), but in contrast to LDA may be considered non-parametric. In SVM, the goal is to find the hyperplane in a high-dimensional space that represents the largest margin between any two instances (support vectors) of two classes of training-data points, or that maximizes a related function if they cannot be separated. Non-linear versions of SVM are devised using a so-called kernel similarity function (Cortes and Vapnik, 1995).

#### 3.3.4. Similarity Matrices and Related Kernel Methods

Some applications of microbiome association testing have compared similarity matrices across features to similarity of traits (Zhao and Shojaie, 2016). A closely-related approach is to first compute principal component (PC) scores, which may be obtained from OTU sample-sample correlation matrices (Zhou et al., 2018), and to use these PC scores as trait predictors. Kernel-penalized regression, an extension of PCA, was utilized by Randolph et al. (2018). in their microbiome data analysis. They applied a significance test for their graph-constrained estimation method, called Grace (Zhao and Shojaie, 2016), to test for association between microbiome species and their trait. However, trait prediction is not available in their software.

#### 3.3.5. *k*-Nearest Neighbors (*k*-NN)

Training samples are vectors in a multi-dimensional space, each with a class label or continuous trait value. For discrete traits, a test sample is assigned the label which is most frequent among the *k* training samples nearest to that point (Figure 1B). Euclidean distance or correlation coefficients are the most commonly used distance metrics. For continuous traits, a weighted average of the *k* nearest neighbors is used, sometimes weighted (e.g., by the inverse of their distance from the new point).

#### 3.3.6. Random Forests

Random forests (Breiman, 2001) are an increasingly used method, extensively applied in many different fields, including computational biology and genomics (Statnikov et al., 2013) The building block of a “forest” is a decision tree, which uses features and associated threshold values to successively split the samples into groups that have similar *y* values. This process is repeated until the total number of specified nodes is reached. An ensemble of decision trees (or regression trees for continuous *y*) is built by performing bootstrapping on the dataset and averaging or taking the modal prediction from trees (a process known as “bagging”)(Figure 1C), with subsampling of features used to reduce generalization error (Ho, 1995). An ancillary outcome of the bootstrapping procedure is that the data not sampled in each bootstrap (called “out of bag”) can be used to estimate generalization error, as an alternative to cross-validation.

#### 3.3.7. Gradient Boosting

Gradient boosting for decision trees refers to a process of ensemble modeling by averaging predictions over decision trees (learners) of fixed size (Friedman, 2001). As with other forms of boosting, the process successively computes weights for the individual learners in order to improve performance for the poorly-predicted samples. Following observations that boosting can be interpreted as a form of gradient descent on a loss function (such as $\underset{i}{\sum }{\left(y_{i}-{ŷ}_{i}\right)}^{2}$), gradient tree boosting successively fits decision trees on quantities known as “pseudo-residuals” (Friedman, 2002) for the loss function (Figure 1C).

#### 3.3.8. Neural Networks

Neural networks refer to an interconnected feed-forward network of nodes (“neurons”) with weights attached to each edge in the network, which allows the network to form a mapping between the inputs *X* and the outcomes *y* (Ditzler et al., 2015a). Each neuron *j* receiving an input *p*_*j*_(*t*) from predecessor neurons consists of the following components: an activation *a*_*j*_(*t*), a threshold θ_*j*_, an activation function *f* that computes the new activation at a given time *t* + 1, and an output function *f*_*out*_ computing the output from the activation. These networks contain either one or many hidden layers, depending on the network type (Figure 1D). For microbiome data, the input layer is the set of OTUs, with separate neurons for each OTU. Hidden layers use backpropagation to optimize the weights of the input variables in order to improve the predictive power of the model. The total number of hidden layers and number of neurons within each hidden layer are specified by the user. All neurons from the input layer are connected to all neurons in the first hidden layer, with weights representing each connection. This process continues until the last hidden layer is connected to the output layer. A bias term is also added in each step, which can be thought of as analogous to the intercept of a linear model. The output layer are predictions based on the data from the input and hidden layers. In most cases, having just one hidden layer with one neuron is reasonable to fit the model.

### 3.4. Measures of Prediction Accuracy: The AUC and Prediction *R*

For predictions ŷ of binary traits, the receiver operating characteristic (ROC) curve plots the true positive rate (TPR) against the false positive rate (FPR) at various threshold settings. The true-positive rate is also known as sensitivity, or a probability of detection. The area under the ROC curve (AUC) is the most common measure of prediction accuracy for binary traits, and ranges from 0.5 (no better than chance) to 1.0 (perfect discrimination). In practice, the empirical AUC can be <0.5, in which case we conclude that the prediction procedure has no value. Note that the AUC is invariant to monotone transformations of ŷ.

The prediction Pearson correlation (*R*) between cross-validated predicted and actual *y* values is a commonly-used standard of accuracy for continuous traits, although many procedures are designed to minimize the mean-squared prediction error $\underset{i}{\sum }{\left(y_{i}-{ŷ}_{i}\right)}^{2}$. *R* ≤ 0 corresponds to no predictive value, and *R* = 1 to perfect prediction. We advocate *R* as a criterion because it is simple and applicable to many prediction procedures. Some prediction procedures may have an offset or proportional bias in prediction that may harm the mean-squared error, even if *R* is favorable. A *post-hoc* linear rescaling of the prediction to “fix” any such bias is straightforward, and we find it simplest to directly use *R* for comparison.

In the real data analyses below, the predicted ŷ represent average predictions over all cross-validation rounds, so the AUC and *R* values were computed directly on the resulting predictions. Importantly, the use of cross-validation provides for each dataset a measure of actual performance of a prediction method, without relying on theoretical considerations, simulations, or restrictive assumptions that may not be applicable with real data.

## 4. Data Used for Comparisons

### 4.1. A Literature Review

We conducted a literature review of published host-trait microbiome prediction studies that used cross-validation and reported a measure of prediction accuracy. We conducted a literature review of published host-trait microbiome prediction studies that used cross-validation and reported a measure of prediction accuracy. A full table appears in the Supplement, including links to each of the 18 studies with 54 reported datasets represented. As different studies used vastly different protocols for OTU generation and preprocessing, for this main paper we focused on the 17 reported datasets that compared at least two competing measures of prediction accuracy. As different studies used vastly different protocols for OTU generation and preprocessing, for this main paper we focused on the 17 reported datasets that compared at least two competing measures of prediction accuracy. All of the datasets were using human hosts, except for Rousk et al. (2010) (where pH in soil samples was the “trait”) and Moitinho-Silva et al. (2017), where microbial abundance in sponges was the trait.

### 4.2. Analyses of Data Using Competing Methods

In addition, we evaluated the supervised learning methods ourselves using datasets from MicrobiomeHD (https://github.com/cduvallet/microbiomeHD), a standardized database of human gut microbiome studies in health and disease. This database includes publicly available 16S rRNA data from published case-control and other studies and their associated patient metadata. The MicrobiomeHD database and original publications for each of these datasets are described in Duvallet et al. (2017). Raw sequencing data for each study was downloaded and processed through a standardized pipeline.

For our analyses, we analyzed four traits (three binary and one continuous) from three datasets with varying sample sizes and initial numbers of OTUs: (1) The Singh et al. (2015) data set, containing 201 EDD (enteric diarrheal disease) cases vs. 82 healthy controls with 1, 325 OTUs. (2) The Vincent et al. (2013) data set, with 25 CDI (Clostridium difficile infection) cases vs. 25 healthy controls and 763 OTUs. (3a) The Goodrich et al. (2014) dataset, which categorized the hosts into 135 obese cases vs. 279 controls, based on body mass index (BMI), with a total of 11, 225 OTUs. In this dataset, individuals came from the TwinsUK population, so we included only one individual from each twin-pair. (3b) The same Goodrich et al. (2014) dataset, but using BMI directly as a continuous phenotype for the same 414 individuals. The microbiome samples for each dataset were obtained from stool, and we analyzed one sample per individual throughout.

Following the filtering recommendations applied by Duvallet et al. (2017), we removed samples with fewer than 100 reads and OTUs with fewer than 10 reads. We also removed OTUs which were present in <1% of samples from the Vincent et al. (2013), Ross et al. (2015), and Singh et al. (2015) datasets, and <5% of samples from the Goodrich et al. (2014) datasets, since it contained many more OTUs. Then we scaled the datasets by calculating the relative abundance of each OTU, dividing its value by the total reads per sample.

In our primary analysis, we tested the relative abundances of the microbiome data at the OTU level. We also ran analyses in which OTUs were collapsed to the genus level by summing their respective relative abundances, discarding any OTUs which were un-annotated at the genus level. Finally, we ran the hierarchical feature engineering (HFE) algorithm introduced by Oudah and Henschel (2018) which results fewer informative features, including individual OTUs and aggregated elements of the taxonomy.

We performed 100 rounds of 5-fold cross-validation for each supervised method, using different random splits for each round. For binary traits, the estimated group probability $\hat{P}\left(Y=1|X\right)$ was used to estimate the group assignment. These estimates were further averaged over the cross-validation rounds. Performance was evaluated using the *AUC*. For continuous traits, the direct estimate ŷ was used, averaged over cross-validations, with performance criterion *R*.

R code for the comparisons is available at https://sites.google.com/ncsu.edu/zhouslab/home/software?, and here we list the packages and settings used. Five-fold cross-validation was used throughout, and we additionally checked for plausibility. For example, the out-of-bag accuracy estimates from the random forest procedure were compared to our cross-validated estimates and shown to match closely. All machine learning methods were used for each dataset as applicable (for example, *LDA* was applicable only for the discrete trait datasets). All predictions used probability estimates for the discrete traits. The random forest method used randomForest with ntree=500, mtry=sqrt(ncol(X)). The gradient boosting (Gboost) decision-tree approach used xgboost, with nrounds=10 and objective= “binary:logistic” for the discrete trait. For the decision tree method, aspects, such as tree depth used default values. The Lasso, Ridge, and Elastic Net approaches used the package and method glmnet, with lambda=seq(0,1,by=0.1). The *k*-NN approach used caret with *k* = 5 and default (equal) neighbor weighting. The neural net used neuralnet with hidden=1, linear.output=F. Linear discriminant analysis used the lda package with tol=0.

## 5. Results

Table 1 shows the comparative results of 17 datasets analyzed with numerous prediction methods. The results for discrete traits were presented as AUC, accuracy, or balanced accuracy, but in all instances higher values reflect better performance. Although not all methods were represented in each study, some general conclusions can be made. When random forests were applied, they were either the most accurate or competitive [with the exception of Nakano (2018)] (Nakano et al., 2018). Various forms of neural networks often performed well, although there is some question whether the tuning complexity is warranted. An exception is Rousk (2010) as analyzed by Ditzler et al. (2015b), in which some neural networks (perceptions) performed especially well, but the sample size was small *n* = 22. In the datasets analyzed by Ditzler et al. (2015b), the complexity and number of nodes in neural networks showed little consistent relationship to performance. Most of the studies used some form of higher-level OTU aggregation, sometimes as high as the phylum level.

**Table 1:** Review of published prediction accuracy comparisons.

**Paper**	**Dataset**	**Trait**	**Samples**	**Cases**	**Controls**	**Taxa**	**Level**	**Method**	**Metric**	**Value**
Pasolli et al., 2016	Qin et al., 2014	Liver cirrhosis	232	118	114	542	Species	Random forest	AUC	0.95
								SVM	AUC	0.92
								Elastic net	AUC	0.91
								Lasso	AUC	0.88
	Zeller et al., 2014	Colorectal cancer	121	48	73	503	Species	Random forest	AUC	0.87
								SVM	AUC	0.81
								Elastic net	AUC	0.79
								Lasso	AUC	0.73
	Qin et al., 2010	IBD	110	25	85	443	Species	Random forest	AUC	0.89
								SVM	AUC	0.86
								Elastic net	AUC	0.83
								Lasso	AUC	0.81
	Le Chatelier et al., 2013	Obesity	253	164	89	465	Species	Random forest	AUC	0.66
								SVM	AUC	0.65
								Elastic net	AUC	0.64
								Lasso	AUC	0.60
	Qin et al., 2012	Type II diabetes	344	170	174	572	Species	Random forest	AUC	0.74
								SVM	AUC	0.66
								Elastic net	AUC	0.70
								Lasso	AUC	0.71
	Karlsson et al., 2013	Type II diabetes	96	53	43	381	Species	Random forest	AUC	0.76
								SVM	AUC	0.66
								Elastic net	AUC	0.60
								Lasso	AUC	0.54
Johnson et al., 2016		Post-mortem interval (PMI)	67	NA	NA	52	Phylum	Ridge	Error rate	0.46
						52	Phylum	Elastic net	Error rate	0.48
						3,130	Species	Lasso	Error rate	0.49
						52	Phylum	SVM	Error rate	0.50
						3,130	Species	Ridge	Error rate	0.51
						3,130	Species	Elastic net	Error rate	0.52
						52	Phylum	Lasso	Error rate	0.52
Ditzler et al., 2015b	Rousk, 2010	Soil pH (low/medium/high)	22	NA	NA	500	Various	Recursive neural network (RNN) (50)	Error rate	0.15
								Deep belief network (DBN) (500)	Error rate	0.08
								Deep belief network (DBN) (750)	Error rate	0.08
								Random forest	Error rate	0.15
								Multi-layer perceptron Neural network (MLPNN) (500)	Error rate	0.00
	Caporaso et al., 2011	Host gender	1,967	NA	NA	500	various	Recursive neural network (RNN) (250)	Error rate	0.15
								Recursive neural network (RNN) (500)	Error rate	0.19
								Deep belief network (DBN) (250)	Error rate	0.24
								Deep belief network (DBN) (500)	Error rate	0.24
								Random forest	Error rate	0.03
								Multi-layer perceptron neural network (MLPNN) (500)	Error rate	0.08
	Caporaso et al., 2011	Three body sites	1,967	NA	NA	500	Various	Recursive neural network (RNN) (250)	Error rate	0.17
								Recursive neural network (RNN) (500)	Error rate	0.16
								Deep belief network (DBN) (250)	Error rate	0.03
								Deep belief network (DBN) (500)	Error rate	0.03
								Random forest	Error rate	0.01
								Multi-layer perceptron neural network (MLPNN) (500)	Error rate	0.01
Reiman et al., 2017	Caporaso et al., 2011	Three body sites	1,967	NA	NA	1,706	Various	Recursive neural network (RNN) (250)	Accuracy	0.83
								Recursive neural network (RNN) (500)	Accuracy	0.84
								Deep belief network (DBN) (250)	Accuracy	0.97
								Deep belief network (DBN) (500)	Accuracy	0.97
								Multi-layer perceptron Neural network (MLPNN) (500)	Accuracy	0.99
								Random forest	Accuracy	0.99
								Convolutional neural Network (CNN-1D)	Accuracy	0.95
								Convolutional neural Network (CNN-2D)	Accuracy	0.99
Moitinho-Silva et al., 2017		Microbial abundance from sponges (high/low)	1,232	NA	NA	30	Phylum	random forest	Accuracy	0.97
								Adaptive boosting (AdaBoost)	Accuracy	0.95
						76	Class	Random forest	Accuracy	0.95
								Adaptive boosting (AdaBoost)	Accuracy	0.91
						2,322	Various	Random forest	Accuracy	0.50
								Adaptive boosting (AdaBoost)	Accuracy	0.91
Ai et al., 2017		Colorectal cancer (CRC)	141	42	99	1,171	Species	Bayes net	AUC	0.93
								Random forest	AUC	0.94
								Logistic	AUC	0.98
			141	53	88	783	Species	Bayes net	AUC	0.86
								Random forest	AUC	0.86
								Logistic	AUC	0.71
Wu et al., 2018		Three diseases	806	423	383	300	Genus	Logistic	F1	0.91
								k-nearest neighbor	F1	0.86
								Random forest	F1	0.83
								SVM	F1	0.91
								Gradient boosting	F1	0.87
								Adaptive boosting	F1	0.90
NAkano et al., 2018		Oral malodour	90	45	45	37	Genus	SVM	Accuracy	0.79
								Deep learning	Accuracy	0.97
Asgari et al., 2018	HMP	Five body sites	1,192	NA	NA	20,589	Various	Random forest	F1	0.89
								SVM	F1	0.85
	Gevers et al., 2014	Crohn's disease	1,359	731	628	9,511	Various	Random forest	F1	0.74
								SVM	F1	0.68

**Figure 2:**
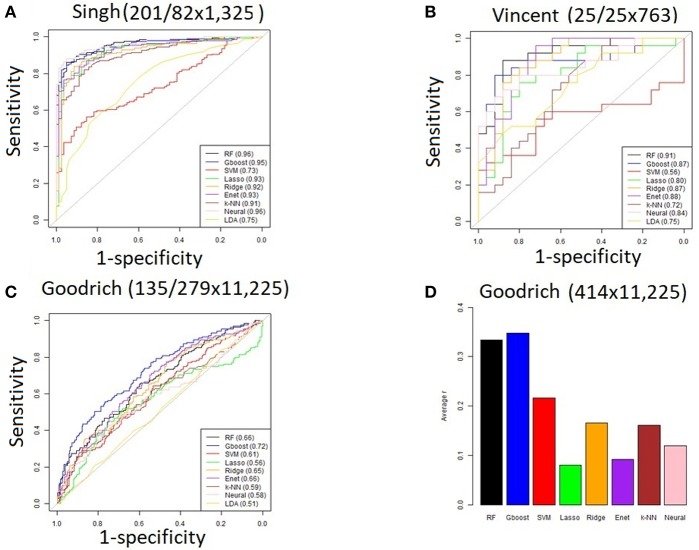
**(A–C)** ROC curves for each machine learning method using all OTUs. The AUC values are shown in the legend. The size of each dataset (# cases/controls ×# OTUs) is shown in the title. **(D)** Bar graph showing the average Pearson correlation (R) between predicted and actual BMI in the Goodrich dataset, using BMI as a continuous trait.

For the three discrete traits, we plotted one ROC curve from each machine learning method (Figures 2A–C). The size of each dataset (number of cases/controls X number of OTUs) is shown in the title. Random forest (RTF) and Gradient boosted trees (Gboost) performed well (AUC >0.85) in predicting cases and controls in the Singh and Vincent datasets. Lasso, ridge, elastic net (Enet), *k*-nearest neighbors (k-NN), and Neural Networks (Neural) performed well in the Singh dataset only. Generally, linear SVM and LDA performed less well, and SVM demonstrated close to chance performance in the Vincent dataset.

Summarizing the results after using BMI as a continuous trait in the Goodrich dataset, the bar graph (Figure 2D) shows the average Pearson correlation between the predicted and actual BMI after 100 iterations of each method. Here again the two decision tree models performed best, although all correlations *R* were <0.4.

**Figure 3:**
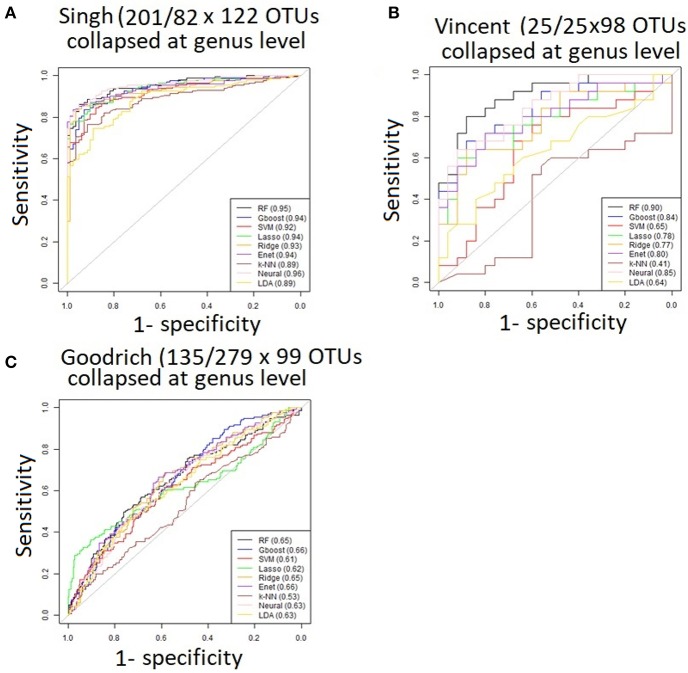
ROC curves after collapsing OTUs to the genus level **(A)** the Singh dataset, **(B)** the Vincent dataset, and **(C)** the Goodrich dataset.

**Figure 4:**
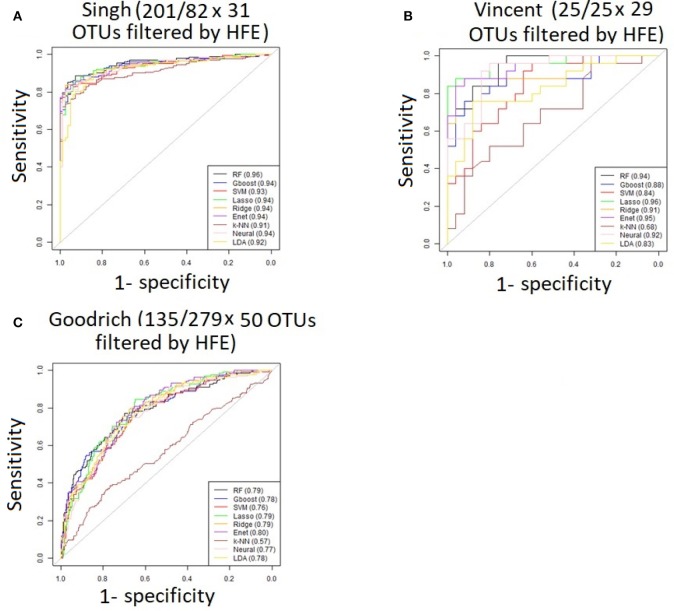
ROC curves after applying the HFE method to select a subset of informative features **(A)** Singh dataset, **(B)** Vincent dataset, **(C)** Goodrich dataset.

**Figure 5:**
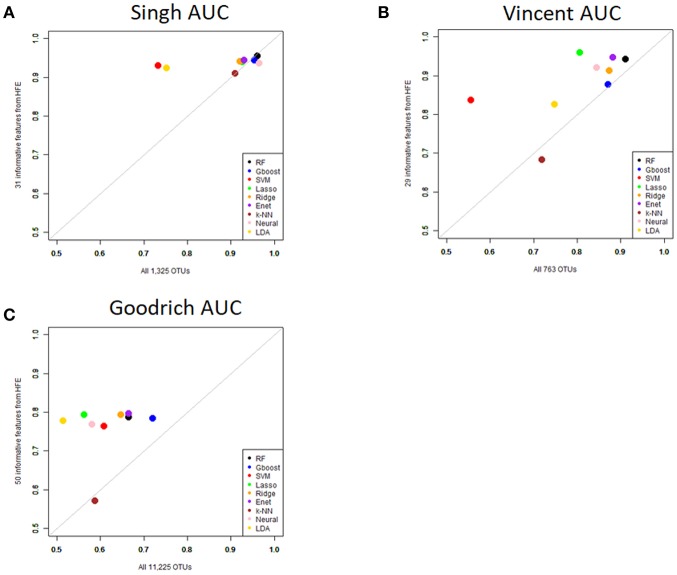
Scatterplot comparing the average AUCs between the full dataset and the HFE subset. **(A)** Singh dataset, **(B)** Vincent dataset, **(C)** Goodrich dataset.

Performance was generally poor for the Goodrich dataset, which also included a large number of OTUs, which presents a challenge in feature selection. We computed the ROC curves for each dataset after collapsing the OTUs to the genus level (Figure 3) and after applying the HFE method to select a subset of informative features (Figure 4). Then we compared the AUCs between the datasets which used all OTUs and those that used only HFE-informative features (Figure 5).

As an overall summary, collapsing to the genus level brought some improvement to the poorer perform prediction methods in the Singh et al. (2015) dataset, and few other broad patterns were apparent. In contrast, the use of cross-validated HFE produced a great improvement in AUC in most instances (Figure 4). For the Goodrich et al. (2014) and Singh et al. (2015) datasets, most methods were improved and brought to similar AUC values. For the Vincent dataset, again most prediction methods were improved by HFE feature-reduction, but the results were less uniform. Another pattern that is apparent in the scatterplots, perhaps expected, is that HFE brought diminishing returns for methods that already perform well. The one prediction method that was not improved demonstrably by HFE was *k*-NN (with *k* = 5).

## 6. Discussion

We have presented a tutorial overview of the most commonly-used machine learning prediction methods in microbiome host trait prediction. Although a large number of approaches have been used in the literature, some relative simple and clear conclusions can be made. Decision tree methods tended to perform well, and in the published literature similar results were achieved by neural networks and their variants. In our analysis, the HFE OTU feature reduction method brought a substantial performance improvement for nearly all methods. In addition, after such feature reduction most methods performed more similarly. We conclude that this finding accords with the fact that the distinction between sparse and non-sparse methods is less dramatic after feature reduction. We hope that the tutorial, review, and available code are useful to practitioners for host trait prediction.

For more advanced topics, we point the reader to analysis of microbiome time series data, using techniques, such as MDSINE (Bucci et al., 2016), which uses dynamical systems inference to estimate and forecast trajectories of microbiome subpopulations. Other uses of dynamical systems have concentrated mainly on observable phenotypes/experiemental conditions, rather than using microbiome status for prediction (Brooks et al., 2017). In addition, the use of co-measured features, such as metabolites (Franzosa et al., 2019), offers potentially useful information for integrative analyses. As another example of the use of ancillary information, an intriguing approach has also been used to predict biotransformation of specific drugs and xenobiotics by gut bacterial enzymes (Sharma et al., 2017). We also note that our review/tutorial has for clarity placed feature engineering, which may be viewed as a form of statistical regularization, as a separately-handled issue from the penalized prediction modeling. Some modern sparse regression and kernel modeling methods seek additional predictive ability by combining feature regularization and prediction in a single step, e.g., Xiao et al. (2018).

## Author Contributions

Y-HZ is the leader of this review study. Her contribution includes writing the manuscript, designing the data analysis, summarizing the result, and software management. PG is responsible for the manuscript writing, implementation of analysis, results summary, and code summary.

### Conflict of Interest Statement

The authors declare that the research was conducted in the absence of any commercial or financial relationships that could be construed as a potential conflict of interest. The handling editor and reviewer HM declared their involvement as co-editors in the Research Topic, and confirm the absence of any other collaboration.

## Supplementary Material

Supplementary Table 1 Full table of published prediction accuracies.
